# Hepatitis E Virus Seroprevalence and Associated Risk Factors in Pregnant Women Attending Antenatal Consultations in Senegal

**DOI:** 10.3390/v14081742

**Published:** 2022-08-09

**Authors:** Abou Abdallah Malick Diouara, Seynabou Lo, Cheikh Momar Nguer, Assane Senghor, Halimatou Diop Ndiaye, Noël Magloire Manga, Fodé Danfakha, Sidy Diallo, Marie Edouard Faye Dieme, Ousmane Thiam, Babacar Biaye, Ndèye Marie Pascaline Manga, Fatou Thiam, Habibou Sarr, Gora Lo, Momar Ndour, Sébastien Paterne Manga, Nouhou Diaby, Modou Dieng, Idy Diop, Yakhya Dieye, Coumba Toure Kane, Martine Peeters, Ahidjo Ayouba

**Affiliations:** 1 Groupe de Recherche Biotechnologies Appliquées & Bioprocédés environnementaux (GRBA-BE), École Supérieure Polytechnique (ESP)–Université Cheikh Anta Diop, Dakar-Fann, Dakar 5085, Senegal; 2 Laboratoire Bactériologie Virologie, Centre Hospitalier Régional de Saint-Louis, Saint-Louis, Senegal; 3 Unité de Formation et de Recherche Science de la Santé (UFR 2S)–Université Gaston Berger, Saint-Louis 234, Senegal; 4 Laboratoire de Bactériologie Virologie CHU Aristide le Dantec–Université Cheikh Anta Diop, Dakar-Fann, Dakar 7325, Senegal; 5 Service des Maladies Infectieuses Hôpital de la Paix de Ziguinchor, Ziguinchor, Senegal; 6 Unité de Formation et de Recherche Science de la Santé–Université Assane Seck de Ziguinchor, Ziguinchor, Senegal; 7 District Sanitaire–Région Médicale de Kédougou, Kédougou, Senegal; 8 Service Hépato-Gastroentérologie, Centre Hospitalier Régional de Saint-Louis, Saint-Louis, Senegal; 9 Clinique Gynécologique et Obstétricale du Centre Hospitalier Universitaire Nationale Aristide le Dantec–Université Cheikh Anta Diop, Dakar-Fann, Dakar 3001, Senegal; 10 Centre de Santé Gaspard KAMARA, Dakar 3370, Senegal; 11 Institut de Recherche en Santé, de Surveillance Épidémiologique et de Formation (IRESSEF)–Pole Urbain Diamniadio, Diamniadio 7325, Senegal; 12 Laboratoire de Traitement des Eaux Usées (LATEU) de l’Institut Fondamental d’Afrique Noir (IFAN)–Université Cheikh Anta Diop, Dakar-Fann, Dakar 206, Senegal; 13 Laboratoire d’Analyses et Essais, École Supérieure Polytechnique (ESP)–Université Cheikh Anta Diop, Dakar-Fann, Dakar 5085, Senegal; 14 Laboratoire d’Imagerie Médicale et BioInformatique, École Supérieure Polytechnique (ESP)–Université Cheikh Anta Diop, Dakar-Fann, Dakar 5085, Senegal; 15 Université Sine Saloum El Hadj Ibrahima Niass (USSEIN), Kaolack 55, Senegal; 16 Recherches Translationnelles sur le VIH et Maladies Infectieuses, Université de Montpellier/INSERM U1175, Institut de Recherche pour le Développement, 34394 Montpellier, France

**Keywords:** hepatitis E, associated risk factors, pregnant women, environment, prevention, Senegal

## Abstract

In West Africa, research on the hepatitis E virus (HEV) is barely covered, despite the recorded outbreaks. The low level of access to safe water and adequate sanitation is still one of the main factors of HEV spread in developing countries. HEV infection induces acute or sub-clinical liver diseases with a mortality rate ranging from 0.5 to 4%. The mortality rate is more alarming (15 to 25%) among pregnant women, especially in the last trimester of pregnancy. Herein, we conducted a multicentric socio-demographic and seroepidemiological survey of HEV in Senegal among pregnant women. A consecutive and non-redundant recruitment of participants was carried out over the period of 5 months, from March to July 2021. A total of 1227 consenting participants attending antenatal clinics responded to a standard questionnaire. Plasma samples were collected and tested for anti-HEV IgM and IgG by using the WANTAI HEV-IgM and IgG ELISA assay. The overall HEV seroprevalence was 7.8% (n = 96), with 0.5% (n = 6) and 7.4% (n = 91) for HEV IgM and HEV IgG, respectively. One of the participant samples was IgM/IgG-positive, while four were declared indeterminate to anti-HEV IgM as per the manufacturer’s instructions. From one locality to another, the seroprevalence of HEV antibodies varied from 0 to 1% for HEV IgM and from 1.5 to 10.5% for HEV IgG. The data also showed that seroprevalence varied significantly by marital status (*p* < 0.0001), by the regularity of income (*p* = 0.0043), and by access to sanitation services (*p* = 0.0006). These data could serve as a basis to setup national prevention strategies focused on socio-cultural, environmental, and behavioral aspects for a better management of HEV infection in Senegal.

## 1. Introduction

Hepatitis E is geographically a very heterogeneously distributed disease, being is present in both developed and developing countries. Hepatitis E virus (HEV) is a nonenveloped and quasi-enveloped virus with an icosahedral capsid. Its genomic structure is a single stranded positive-sense RNA containing three open reading frames (ORF 1–3). The second (ORF 2) is the one encoding for the protein of the viral capsid targeted by neutralizing antibodies directed against HEV [1,2,3].

To date, HEV genetic diversity shows eight different genotypes (HEV-1 to HEV-8), all belonging to *Paslahepevirus balayani* and infecting mammals [4,5,6]. Of these genotypes, HEV-1, HEV-2, HEV-3, and HEV-4 are most commonly associated with HEV infection in humans. Genotypes HEV-1 and HEV-2 are restricted to humans, whereas genotypes HEV-3 and HEV-4 have a broader host range and are zoonotic. Without being exclusive, genotypes 1 and 2 mainly co-circulate in tropical and subtropical countries in Asia and Africa, while genotype 3 has worldwide distribution. In contrast, HEV genotype 4 is found exclusively in Asia [2,4,5,7,8].

Transmission is essentially by the fecal–oral route, causing generally asymptomatic infection [1,9,10]. To some extent, cases of mother-to-child transmission have also been reported [11,12,13,14], the zoonotic transmission being linked to ingestion of raw shellfish and undercooked pork meat [15,16,17,18,19,20]. Studies also report cases of infections in humans with *Rocahepevirus ratti* (rat hepatitis E virus) [21,22,23]. Ingestion of fruits and vegetables contaminated by irrigation water has also been described as a route of contamination by HEV [24,25]. More generally, through contaminated water and crops, the environmental aspects seem to be a significant vector for the spread of HEV in developing countries [1,26,27].

Most infections are self-limited acute hepatitis in immunocompetent subjects. However, it can become severe with very high mortality rates in specific population groups, including pregnant women and immunosuppressed people [28,29,30,31,32]. Indeed, the mortality rate in the general population is around 0.5 to 4%, while pregnant women are more likely to develop complicated forms of the disease that can lead to mortality rates ranging from 20 to 25% [11,28,29,33]. High neonatal mortality and morbidity have also been reported [13,14,28,34]. Therefore, HEV infection is considered as a promoting factor that can lead to hepatocellular carcinoma [33]. The WHO estimates that 20 million HEV infections and more than 3.3 million acute cases of hepatitis E are detected per year, with an estimated death of 56,600 cases [35].

In developed countries, the seroprevalence of anti-HEV IgG was estimated to range from 7% to 21% [5]. Kim et al., in their systematic review from studies conducted in Africa, reported at least 17 outbreaks since 1979 in 28 out of 54 countries, causing a reported 35,300 cases with 650 deaths [36]. Recent outbreaks of waterborne hepatitis E have been reported with high levels of endemicity. More specifically, some countries such as Uganda and Niger, as well as neighboring countries of the Lake Chad Basin including Nigeria, Chad, South Sudan, and North Cameroon, seem to be more affected [10,37,38,39,40]. The number of cases listed, including epidemic episodes, seems to be largely underestimated [36]. In pregnant women, reported seroprevalences of HEV were 16.19%, 11.6%, and 83.3% in Benin [41], Burkina Faso [42], and Egypt [43], respectively. The carriage of anti-HEV antibodies in healthy adults is estimated at 93%, while it was lower (43%) in healthcare workers in Nigeria [44] and varies from 0 to 84% in other African countries [36].

In year 2014, a localized epidemic was declared in the gold-bearing area of Kédougou located in the south-eastern region in Senegal. Local health authorities reported 19 deaths, and almost all of the infected individuals came from traditional gold panning sites, which concentrate a community of several workers from African countries, especially those bordering Senegal [45]. Since then, very little epidemiological data has been available at the national level. It should be noted that the diagnosis of hepatitis E is not routinely performed, even less in pregnant women with symptoms that would suggest a potential infection.

According to the 2018 Demographic and Health Survey report [46], the coverage of prenatal care is estimated at 98% in Senegal. Thus, almost all women aged 15–49 who delivered a child received prenatal care from a qualified provider, including midwives (91%). Six out of ten women made at least four prenatal visits (59%), and in 64% of cases, the first visit took place before the fourth month of pregnancy [46,47]. However, disparities were mentioned according to place of residence, 71% in urban areas against 50% in rural areas [46].

Furthermore, beyond the health, economic, and environmental concerns, the issue of hepatitis E in West Africa is poorly covered, as evidenced by the very limited number of scientific studies [36,48]. From a strategic point of view, obtaining new epidemiological data is necessary and will make it possible to fill this gap. For this, the main objective was to determine the seroprevalence and associated risk factors with hepatitis E virus infection in pregnant women attending antenatal consultations in Senegal.

## 2. Materials and Methods

### 2.1. Study Sites, Sampling and Data Collection

**Figure 1:**
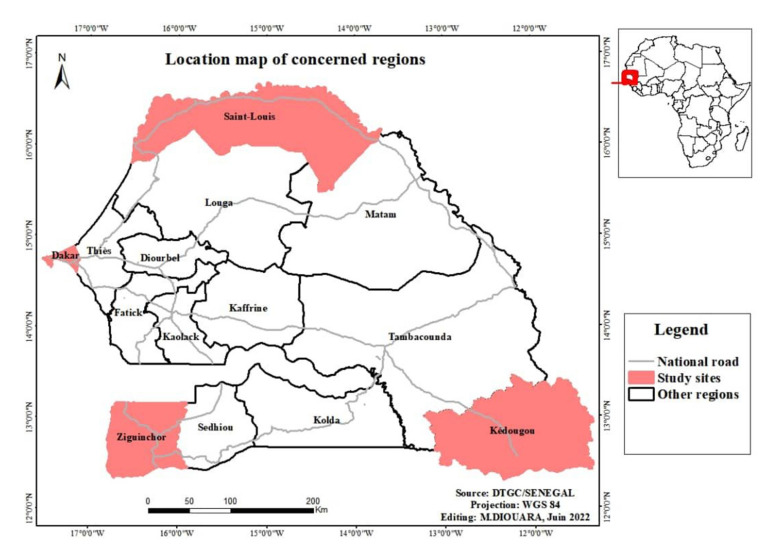
Map of Senegal, with indication of the geographical sites of the study.

This prospective and multi-site study is part of a research program on the epidemiology of hepatitis E leaded by the GRBA-BE (Groupe de Recherche Biotechnologies Appliquées et Bioprocédés Environnementaux)/JEAI EPIVHE (Jeune Équipe Associée à l’IRD (Institut de Recherche pour le Développement) EPIVHE (Environnement et Epidémiologie du virus de l’hépatite E)) and his collaborators. Except for Dakar, the capital city of Senegal that housed two sites, all the three other regions had only one (Figure 1). Regardless of geographic region, all inclusion sites are located in urban areas with relatively good attendance according to antenatal care providers. The sample size was estimated by taking into account data on antenatal care coverage from the Demographic and Health Survey [46] (described above) including information collected directly from participating sites (the number of antenatal care visits recorded during two years before the study). Therefore, the sampling plan forecasted around 20 to 30%, with a minimum of 200 pregnant women per site, or 1000 participants over the enrolment period.

The inclusion criteria were pregnant woman from four weeks of amenorrhea confirmed by a pregnancy test and/or ultrasound, aged 18 years or over, resident for at least 6 months in the targeted localities, and consenting to participate in the study. Those with acute alcoholic hepatitis or drug-induced hepatitis and/or non-consenting were not included. On all the sites, a consecutive and non-redundant recruitment of participants was carried out over the period from March to July 2021. Socio-demographic and other relevant information to the study were collected with standardized survey forms and through individual and anonymous interviews. This consisted of collecting the address of the place of residence including trips over the past 12 months, access to sanitation and safe water supply services, individual and community hygiene (systematic hand washing after using the toilet, disinfection and rinsing of fruits and vegetables before consumption), age, education level, regular income linked to a professional activity, and marital status. The data collected were entered directly into an Excel file.

For each participant, a whole blood sample was taken on EDTA tubes for laboratory analysis. Lymphocyte separation was performed within two hours after collection and the plasma was frozen at −80 °C or stored at −20 °C on site until processing. An individual identification code per site and per patient was assigned to each sample. A written and signed informed consent form was obtained from each participant before the interview and sample collection. Ethical and administrative approvals were also obtained from the Senegalese National Ethics Committee for Health Research (N°000130/MSA/CNRES/Sec) and the Ministry of Public Health and Social Action (N°00000582/MSAS/DPRS/DR).

As the study was carried out in the context of COVID-19 pandemic, in accordance with the recommendations of the local health authorities, our field teams have taken all the necessary measures to prevent and fight against the spread of the SARS-CoV-2 infection.

### 2.2. Anti-HEV Antibody Detection

To detect anti-HEV IgM and IgG, we used enzyme-linked immunosorbent assay (ELISA) from Wantai Biological Pharmacy Enterprise, Beijing, China (Wantai HEV-IgM ELISA and Wantai HEV-IgG ELISA), as per the manufacturer’s instructions. The specific HEV IgM and IgG antibodies were detected by adding plasma samples to ELISA plates precoated with recombinant HEV ORF2 antigens. HEV antibodies, if present in plasma sample, bind to these precoated antigens, which subsequently react with anti-human IgG or IgM conjugated to the enzyme horseradish peroxidase (HRP-conjugate). The reaction was then revealed by the addition of a chromogen (tetramethyl benzidine, TMB). The reported sensitivities and specificities are in the range of 97.10–98.40% for HEV IgM antibodies and 99.08–99.90% for HEV IgG antibodies. In addition, the HEV-IgM represents the best marker for detecting the acute HEV infection, where RT-PCR cannot be performed [49]. Optical density was read using the MICRO READ 1000 ELISA Plate Analyser (Global Diagnostics B, Belgium). The results are calculated by relating each specimen absorbance (A) value to the cut-off value (C.O.) of the plate. For the calculation of the cut-off value (C.O.), it was C.O = Nc + 0.16 and C.O = Nc + 0.26, respectively, for HEV IgG and IgM antibodies. In both cases, Nc = the mean of absorbance value for three negative controls). Index was defined as A/C.O. The tests were declared to be negative if IgM or the IgG index was < 1, positive if the index was ≥ 1, and borderline if the index was = 0.9–1.1.

All samples declared positive in the first tests were re-tested by Wantai HEV-IgM and HEV-IgG ELISA in accordance with the manufacturer’s instructions.

### 2.3. Statistical Analysis

Statistical analysis was performed with JMP^®^ Pro Version 15.0.0 software (SAS Institute Inc., Cary, NC, USA, 1989–2021). To assess the sociodemographic and environmental factors associated with exposure to HEV infection, we performed bivariate analyses. With regard to the data of binary variables whose frequencies were less than 5, chi-squared or Fisher’s exact tests were carried out. For all calculations, the confidence interval was set at 0.95. *p*-values < 0.05 were considered statistically significant.

## 3. Results

A total of 1227 pregnant women attending antenatal consultations were recruited throughout five health facilities across four different geographic regions. In Dakar, the recruitment was carried out at the Obstetrical Gynecology Center of the Aristid Le Dantec hospital (*n* = 50) and at the Gaspard Kamara Health Center (*n* = 116). At the other sites, we recruited 400, 397, and 264 participants, respectively, at the Regional Hospital Center of Saint-Louis, the Health District of Kédougou, and the NEMA Health Center of Ziguinchor. The median age was 25 years (age range 18–50 years). The distribution of age groups showed a greater representation of the 18–23-year-olds with 43%, 24–29-year-olds for 29.1%, followed by the 30–35-year-olds with 18.2%. Participants aged 36 and above represented only 9.7%. Of these, 3.6% (*n* = 45) were aged ≥ 40 years. Despite a low participation rate in Dakar and Ziguinchor localities, the total number of pregnant women enrolled was very satisfactory (*n* = 1227 vs. 1000 participants expected). The overall and site-specific results of the survey relating to hand hygiene, the disinfection of unpackaged fresh fruits and vegetables before consumption and access to safe drinking water and sanitation services, educational level, marital status, and regular income are summarized in Table 1.

In this study, 31.7% of the participants were without instructions, with higher rate observed in the locality of Kédougou (58.7%). Moreover, only 9% of them reached a higher level of education. Unlike the other localities, 25.9% of the participants in the city of Dakar reached a higher level of education. It should be noted that 92% of the participants in this study declared that they were married, and only 18.7% had regular income (salaried or self-employed workers). On this last point, the highest rate was observed in Dakar (45.8%), which contrasts with that noted in Saint-Louis (11.8%).

Overall, 78.4% of pregnant women reported having access to safe drinking water. However, a remarkably low rate was noted in Ziguinchor (37.1%). Indeed, 42.8% of participants reported not having access to sanitation services, including adequate toilets. The level of access for women residing in other localities was relatively acceptable and varied from 91.4% to 100%.

Regarding hand hygiene, overall, more than 90% of participants declared that they systematically washed their hands, especially after using the toilet. In addition, 71.3%, 87.7%, and 100% of the participants, respectively, from Saint-Louis, Kédougou, and Dakar declared that they proceeded to the decontamination of food matrices (fruits and vegetables), particularly those not wrapped and eaten raw. However, among respondents from Ziguinchor, almost 30% said they did not systematically decontaminate fruits and vegetables eaten raw (Table 1).

The overall seroprevalence of HEV was 7.8% with 0.5% (*n* = 6) and 7.4% (*n* = 91) of participants were positive for IgM and IgG antibodies to HEV, respectively. Only one sample was positive for both IgM and IgG. A total of 4 samples were declared indeterminate to anti-HEV IgM, despite having been re-tested according to the WANTAI HEV-IgM and IgG ELISA detection kit manufacturer’s instructions. The observed prevalence rate of HEV varied from one geographic region to another. For anti-HEV IgM, no positive sample was identified in Dakar and Ziguinchor, while it was 0.5 and 1% for Saint-Louis and Kédougou, respectively. The HEV IgG seroprevalence was higher in the regions of Saint-Louis and Kédougou, with 10.5% and 9.6%, respectively, while in Dakar and Ziguinchor, it was 4.2 and 1.5%, respectively. Between sites, the differences observed were statistically significant only for the IgG seroprevalence (*p* = 0.0133) (Table 2).

Furthermore, analysis of the aggregated data suggests a link between the age of the participants and exposition to HEV (*p*-values were 0.0372 and 0.0048 for IgM and IgG, respectively). With regard to age groups, this association is more remarkable among young adults (18–35 years), where more than 80% of infections were observed (Table 2). In addition, marital status (*p* < 0.0001), economic situation (regular income) (*p* = 0.0043), and access to sanitation services (adequate toilets, appropriate wastewater disposal system) (*p* = 0.0006) were significantly associated with exposure to HEV (Table 3).

## 4. Discussion

This study aimed at documenting the seroprevalence of the hepatitis E virus (HEV) in pregnant women attending antenatal consultations in five health facilities distributed in four different geographical regions. The overall HEV seroprevalence found was high (7.8%), with 0.4% (*n* = 6) and 7.4% (*n* = 91) of IgM and IgG, respectively. Otherwise, this overall seroprevalence hides disparities between sites (Table 1). Similar seroprevalence has been reported in other studies conducted in pregnant women, especially in the third trimester in Nigeria [50], among the HIV-1-positive pregnant women in central Africa [32]. Furthermore, our results differ from those of a multi-center study of 398 pregnant women in Ghana, where the seroprevalences of HEV were 12.20 and 0.2%, respectively, for IgG and for IgM [34]. Adjei et al. report higher prevalence of HEV IgM (64.40%) and HEV IgG (35.60), with positivity mainly observed in young adults (20–25 years) [51]. In Ethiopia, a study conducted among pregnant women showed that 359 (42.4%) and 8 (0.9%) were tested positive for anti-HEV IgG and anti-HEV IgM antibody, respectively [52]. HEV infection investigation among patients with acute febrile jaundice in Burkina Faso showed 2.6% and 18.2%, respectively, for anti-HEV IgM and IgG among 900 patients [53].

Overall, an inter-site variability was observed both for the serological markers and for the associated sociodemographic factors (Table 2 and Table 3). Similar results were also reported in a recent review dealing with viral hepatitis E outbreaks in refugees and internally displaced populations in sub-Saharan Africa [27]. It is important to emphasize the differences of the responses related to the questionnaire provided by pregnant women, between sites related to the participation rates and also the completeness or otherwise.

Hight rate of participation was obtained, which could be considered as indicative of good attendance at prenatal care structures. Compared to other regions, Dakar recorded lower participation rates (*n* = 166, 41.5% to what expected). This situation could be linked to the fact that recruitment had taken place in the midst of a crisis due to the COVID-19 pandemic. Dakar, the capital city, concentrated more cases across the country, and therefore its health facilities were rarely visited by the population for fear of contracting COVID-19. This low participation rate coincides with the fact that none of plasma samples tested revealed anti-HEV IgM positivity, an indicator of acute infection, while 7 (4.2%) were positive for anti-HEV IgG antibodies, indicating previous exposure to HEV. Previous studies carried out in Dakar also revealed rare cases of hepatitis E infections [54,55,56]. Access to adequate sanitation services and safe drinking water, including the participants’ level of education (more than 50% reached the level of education equal to or higher than secondary school), could support the low seroprevalence of HEV observed in Dakar. The participant also declared that they systematically wash their hands after using the toilets and disinfect raw fruits and vegetables before consumption. In addition, among them, 45.8% of them had a regular income (Table 1). Similar results have been reported in studies conducted in Tunisia and Turkey, showing that advanced age (>30), promiscuity, lower educational and income levels, and rural residence were correlated with higher anti-HEV IgG-positive values [57,58].

The participation rate was also relatively low in Ziguinchor, where no case of anti-HEV IgM antibody positivity was detected, and the seroprevalence of anti-HEV IgG was 1.5%, the lowest rate of all sites. Besides these reasons mentioned above, to try to explain these results, they are contradictory because 82.2% of the participants had no regular income related to work, 57.2% had no access to safe water, and 42 8% also did not have access to toilets that met sanitary standards. In addition, 18.2% of pregnant women declared that they did not systematically wash their hands with soapy water after using the toilet, and nearly 30% did not systematically decontaminate raw fruits and vegetables before consumption. Thus, this partial result of survey seems contradictory to serological tests obtained in Ziguinchor as for the other remaining sites. The same is true with data from the literature showing a link between these factors and the risk of HEV infection [34,48,52,59].

Except for IgG positivity (10.50%) found in Saint-Louis participants, the positivity rates for anti-HEV IgM (1%) and IgG (9.6%) in pregnant women in Kédougou were higher than those of the other localities. While contexts were different, the data of this study contrast with those reported during the 2014 epidemic. The prevalence rates of IgM and IgG in individuals who were identified in contact with people who tested positive for hepatitis E by RT-PCR and suspected on the basis of symptoms were 38.8% and 27.5% for IgM and IgG, respectively. It should be noted that this study population of the 2014 epidemic had previously tested negative by RT-PCR. This study reports that the risk of exposure was statistically higher in men (77.3%) than in women (22.7%). However, serious cases have been observed mainly in women, particularly those who are pregnant. Moreover, among them, two cases of death due to hepatitis E were noted during this study [45].

Our work also has some limitations. First, the study sites are all located in urban areas. We were also unable to establish a link between seroprevalence of HEV infection and the pregnancy term. Another limitation is the lack of molecular data to confirm acute infection. This aspect is planned in further development of the project.

## 5. Conclusions

With a satisfactory participation rate (122.7%) related to the expected sampling, this seroepidemiological survey confirms the circulation of HEV in Senegal and in particular contributes to a better understanding of hepatitis E virus infection in pregnant women with a national seroprevalence of 7.8%. Our data confirm also that HEV is a poverty-linked infection, as evidenced by the significant association of seroprevalence with regular income and access to sanitation services. Thus, as a means to mitigate this emerging infection, a holistic intervention approach should be adopted.

## Figures and Tables

**Table 1:** Socio-demographic characteristics of pregnant women and HEV markers.

Variable	Study Sites									
	Saint-Louis (*n* = 400)		Dakar (*n* = 166)		Kédougou (*n* = 397)		Ziguinchor (*n* = 264)		All Sites (*n* = 1227)	
	Frequency (%)	Median	Frequency (%)	Median	Frequency (%)	Median	Frequency (%)	Median	Frequency (%)	Median
**Range of age**										
18–23	112 (28)	20	49 (29.5)	21	256 (64.5)	19	111 (42)	20	528 (43)	20
24–29	136 (34)	26	44 (26.5)	27	90 (22.7)	26	87 (33)	26	357 (29.1)	26
30–35	101 (25.3)	32	48 (28.9)	32.5	29 (7.3)	30	45 (17)	32	223 (18.2)	32
36 and above	51 (12.8)	36	25 (15.1)	37	22 (5.5)	38	21 (8)	39	119 (9.7)	38
**Educational level**										
None	62 (15.5)	.	25 (15.1)	.	233 (58.7)	.	69 (26.1)	.	389 (31.7)	.
Primary	170 (42.5)	.	46 (27.7)	.	82 (20.7)	.	77 (29.2)	.	375 (30.6)	.
Secondary	122 (30.5)	.	52 (31.3)	.	75 (18.9)	.	104 (39.4)	.	353 (28.8)	.
Higher	46 (11.5)	.	43 (25.9)	.	7 (1.8)	.	14 (5.3)	.	110 (9)	.
**Marital status**										
unspecified	11 (2.8)	.	0 (0)	.	16 (4.0)	.	4 (1.5)	.	31 (2.5)	.
Single	8 (2)	.	6 (3.6)	.	12 (3)	.	39 (14.8)	.	65 (5.2)	.
Married	381 (95.3)	.	160 (96.4)	.	369 (93)	.	220 (83.3)	.	1130 (92)	.
Divorced or widowed	0 (0)	.	0 (0)	.	0 (0)	.	1 (0.4)	.	1 (0.08)	.
**Regular income (paid work)**										
unspecified	6 (1.5)	.	1 (0.6)	.	36 (9.1)	.	4 (1.5)	.	47 (3.8)	.
Yes	47 (11.8)	.	76 (45.8)	.	63 (15.9)	.	43 (16.3)	.	229 (18.7)	.
No	347 (86.8)	.	89 (53.6)	.	298 (75.1)	.	217 (82.2)	.	951 (77.5)	.
**Access to safe water supply services**										
unspecified	2 (0.5)	.	0 (0)	.	24 (6)	.	5 (1.9)	.	31 (2.5)	.
Occasionally	0 (0)	.	0 (0)	.	0 (0)	.	10 (3.8)	.	10 (0.8)	.
Yes	387 (96.8)	.	166 (100)	.	311 (78.3)	.	98 (37.1)	.	962 (78.4)	.
No	11 (2.8)	.	0 (0)	.	62 (15.6)	.	151 (57.2)	.	224 (18.3)	.
**Access to sanitation services**										
unspecified	1 (0.3)	.	0 (0)	.	12 (3)	.	5 (1.9)	.	18 (1.5)	.
Occasionally	0 (0)	.	0 (0)	.	0 (0)	.	5 (1.9)	.	5 (0.4)	.
Yes	388 (97)	.	166 (100)	.	363 (91.4)	.	141 (53.4)	.	1058 (86.2)	.
No	11 (2.8)	.	0 (0)	.	22 (5.5)	.	113 (42.8)	.	146 (11.9)	.
**Disinfection of food products that are not wrapped and handled by hand (examples: Vegetables. fruits. etc.)**										
unspecified	1 (0.3)	.	0 (0)	.	28 (7.1)	.	3 (1.1)	.	32 (2.6)	.
Occasionally	59 (14.8)	.	0 (0)	.	0 (0)	.	1 (0.4)	.	60 (4.9)	.
Yes	285 (71.3)	.	166 (100)	.	348 (87.7)	.	182 (68.9)	.	981 (79.9)	.
55 (13.8)	.	0	.	21 (5.3)	.	78 (29.5)	.	154 (12.5)	.
**Systematic hand washing**										
unspecified	0 (0)	.	0 (0)	.	16 (4)	.	4 (1.5)	.	20 (1.6)	.
Occasionally	0 (0)	.	0 (0)	.	0 (0)	.	6 (2.3)	.	6 (0.5)	.
Yes	392 (98)	.	166 (100)	.	350 (88.2)	.	206 (78)	.	1114 (90.8)	.
No	8 (2)	.	0 (0)	.	31 (7.8)	.	48 (18.2)	.	87 (7.1)	.
**HEV markers seroprevalance**										
HEV IgM Positive	2 (0.5)	.	0 (0)	.	4 (1)	.	0 (0)	.	6 (0.5)	.
HEV IgG Positive	42 (10.5)	.	7 (4.2)	.	38 (9.6)	.	4 (1.5)	.	91 (7.4)	.

**Table 2:** Variability of HEV IgM and IgG serological markers according to age groups and localities.

	IgM HEV		IgG HEV	
[Age Groups] (%)	*n* (%)	*p*-Value	*n* (%)	*p*-Value
[18,19,20,21,22,23], *n* = 528 (43)	1 (0.18)	0.0372	28 (5.30)	0.0048
[24,25,26,27,28,29], *n* = 357 (29.1)	2 (0.56)		26 (7.28)	
[30,31,32,33,34,35], *n* = 223 (18.2)	2 (0.89)		19 (8.52)	
≥36 years, *n* = 119 (9.7)	1 (0.84)		18 (15.12)	
**Total (*n* = 1227)**	6 (0.48)		91 (7.41)	
**Location (Frequency)**				
Saint-Louis (*n* = 400)	2 (0.50)	0.3293	42 (10.50)	0.0133
Dakar (*n* = 166)	0 (0.00)		7 (4.21)	
Ziguinchor (*n* = 264)	0 (0.00)		4 (1.51)	
Kédougou (*n* = 397)	4 (1.00)		38 (9.57)	
**Total (*n* = 1227)**	6 (0.48)		91 (7.41)	

**Table 3:** Prevalance of HEV IgM and IgG markers and potential associated factors.

		IgM HEV			IgG HEV		
Educational Level	Frequency (%)	*n*	Prevalence	*p*-Value	*n*	Prevalence	*p*-Value
None	389 (31.7)	4	1.03	0.4655	32	8.23	0.4017
Primary	375 (30.6)	1	0.27		30	8	
Secondary	353 (28.8)	1	0.28		25	7.08	
Higher	110 (9)	0	0		4	3.64	
**Marital status**							
unspecified	31 (2.5)	0	0	0.9999	2	6.45	< 0.0001
Single	65 (5.2)	0	0		7	10.77	
Maried	1130 (92)	6	0.53		82	7.26	
Divorced or widowed	1 (0.08)	0	0		0	0	
**Regular income (paid work)**							
unspecified	47 (3.8)	0	0	1	1	2.13	0.0043
Yes	229 (18.7)	1	0.44		12	5.24	
No	951 (77.5)	5	0.53		78	8.2	
**Access to the potable water supply service**							
unspecified	31 (2.5)	1	3.23	0.1958	2	6.45	0.4001
Occasionally	10 (0.8)	0	0		0	0	
Yes	962 (78.4)	4	0.42		78	8.11	
No	224 (18.3)	1	0.45		11	4.91	
**Access to sanitation services (Adequate toilets, appropriate wastewater disposal system)**							
unspecified	18 (1.5)	0	0	1	2	11.11	0.0006
Occasionally	5 (0.4)	0	0		0	0	
Yes	1058 (86.2)	6	0.57		87	8.22	
No	146 (11.9)	0	0		2	1.37	
**Disinfection of food products that are not wrapped and hand-handled (examples: Vegetables, fruits, etc.)**							
unspecified	32 (2.6)	0	0	1	2	6.25	0.5984
Occasionally	60 (4.9)	0	0		6	10	
Yes	981 (79.9)	6	0.61		75	7.65	
No	154 (12.5)	0	0		8	5.19	
**Systematic hand washing**							
unspecified	20 (1.6)	0	0	0.4406	2	10	0.1950
Occasionally	6 (0.5)	0	0		0	0	
Yes	1114 (90.8)	5	0.45		87	7.81	
No	87 (7.1)	1	1.15		2	2.3	

## Data Availability

Not applicable.
